# Vitreomacular Interface: From Anterior to Tangential Traction

**DOI:** 10.1155/2015/197513

**Published:** 2015-09-06

**Authors:** Mario R. Romano, Xavier Valldeperas, John Byron Christoforidis

**Affiliations:** ^1^Department of Neuroscience, University Federico II, Naples, Italy; ^2^Germans Trias i Pujol University Hospital, Barcelona, Spain; ^3^University of Arizona Medical Center, Tucson, AZ 85711, USA

Anomalous posterior vitreous detachment (PVD) plays a major role in the formation of anterior-posterior and tangential epiretinal traction. Advances in the tomographic analysis of vitreous traction vectors have highlighted the selected cases that can benefit from “*parasurgical*” approaches, providing a different perspective for therapeutic strategies. The symptomatic anterior-posterior traction recently gained crescent interest for the application of enzymatic vitreolysis (ocriplasmin, IVO), mainly in small traction associated with full thickness macular hole.

On the other hand, the outer layer of posterior vitreous cortex leads to tangential traction responsible for the formation of macular pucker. As reported by M. R. Romano et al., the presence of vitreoschisis associated with a strong vitreopapillary adhesion of posterior cortex enhances the force of the tangential vector, inducing potential damage to the inner nuclear and outer plexiform layers. They also reported the epiretinal presence of glial fibrillary acidic protein (GFAP), indicating the ability of these cells to proliferate and migrate on the retinal surface. The transdifferentiated epiretinal cells are characterized by a downregulation of GFAP and cytokeratins, while proteins involved in motility and proliferation, such as *α*-smooth muscle actin, are upregulated. Therefore, the GFAP content in epiretinal tissues has been shown to correlate directly with tractional forces and inversely with the clinical contractility.

D. Compera et al. reported interesting histology findings on the presence of GFAP positive cells in lamellar-hole (LMH) associated epiretinal proliferation. The authors hypothesize that the vitreous derived cells, rather than cells of glial origin, may play a relevant role in the pathogenesis of tangential traction. In LMH, differently from macular pucker, the traction is not increased by the contraction of *α*-smooth muscle actin (*α*-SMA), as *α*-SMA-positive myofibroblasts were an infrequent finding in epiretinal proliferation associated with LMH, in their report.

D. Agarwal et al. highlighted the presence of a tangential traction due to the thickened premacular vitreous cortex in diabetic patients. The authors also described a significant relationship between PVD and lack of macular edema, suggesting the importance of the vitreous cortex, embedded with fibroblasts and astrocytes, in the development and progression of diabetic retinopathy. The different adhesion at the interfaces seems to be sustained by factors such as VEGF, CCL2, IL-6, IL-8, and IL-18, also encouraging neovascularization and therefore worsening visual outcome.

Surgical treatment is still the main approach in the management of the tangential tractional membranes. F. Semeraro et al. showed that the pathological vitreoretinal adhesion on the inner limiting membrane (ILM) offers different surfaces on which transdifferentiated glial cells migrate and thereby configures different aspects of traction maculopathies. The removal of ILM has become a routine practice in the surgery of the epiretinal membranes (ERM). However, many recent studies have shown that ILM peeling may cause immediate iatrogenic damage and progressive modification on the underlying inner retinal layers. R. Gelman et al. reported retinal damage induced by ILM peeling including dye-induced toxicity, Müller cell dysfunction, dissociated optic nerve fiber layer, paracentral hole formation, and phototoxic damage. Interestingly, W. J. Mayer et al. reported, in prospective study on 42 eyes, the evidence of mechanical trauma of ERM and ILM peeling due to the use of intraocular forceps that may affect the outer retinal structure. In fact, they observed a significant correlation between the integrity of the ellipsoid zone and retinal sensitivity, overall and in the peeled areas.

M. Asencio-Duran et al. reported that the earliest change in the macula is postoperative swelling of the arcuate retinal nerve fiber layers that appears as hypoautofluorescent arcuate striae on infrared and autofluorescence imaging. Such findings induced by the swelling may disappear in 3 months; however they may potentially cause permanent functional damage in patients already affected by glaucoma.

Finally, vitreomacular adhesion (VMA) can also affect the progression of the age-related macular degeneration (AMD), as reported by E. C. Kang and H. J. Koh. In neovascular AMD, VMA may induce inflammation, decrease in oxygenation, and sequestering of VEGF and other cytokines. Moreover, VMA may also interfere with the therapeutical effects of anti-VEGF treatment.

Considering movement towards future, a more updated classification of the tangential traction is needed, whereas the anterior-posterior traction has been better classified thanks to the recent interest in the enzymatic vitreolysis.

*Mario R. Romano* *Mario R. Romano* *Xavier Valldeperas* *Xavier Valldeperas* *John Byron Christoforidis* *John Byron Christoforidis*

